# Individual patient data meta-analysis of survival data using Poisson regression models

**DOI:** 10.1186/1471-2288-12-34

**Published:** 2012-03-23

**Authors:** Michael J Crowther, Richard D Riley, Jan A Staessen, Jiguang Wang, Francois Gueyffier, Paul C Lambert

**Affiliations:** 1 Centre for Biostatistics and Genetic Epidemiology, Department of Health Sciences, University of Leicester, Adrian Building, University Road, Leicester LE1 7RH, UK; 2 School of Health and Population Sciences, University of Birmingham, Birmingham B15 2TT, UK; 3 The Studies Coordinating Centre, Division of Hypertension and Cardiovascular Rehabilitation, Department of Cardiovascular Research, University of Leuven, Campus Sint Rafaël, Kapucijnenvoer 35, Block D, Box 7001, Leuven BE-3000, Belgium; 4 Department of Epidemiology, Maastricht University, Peter Debyeplein 1, Box 616, Maastricht, MD NL-6200, The Netherlands; 5 Centre for Epidemiological Studies and Clinical Trials, Ruijin Hospital, Shanghai Jiaotong University School of Medicine, Ruijin 2nd Road 197, Shanghai 200025, China; 6 INSERM, CIC201, Lyon F-69000, France; 7 Department of Medical Epidemiology and Biostatistics, Karolinska Institutet, Box 281, Stockholm S-171 77, Sweden

## Abstract

**Background:**

An Individual Patient Data (IPD) meta-analysis is often considered the gold-standard for synthesising survival data from clinical trials. An IPD meta-analysis can be achieved by either a two-stage or a one-stage approach, depending on whether the trials are analysed separately or simultaneously. A range of one-stage hierarchical Cox models have been previously proposed, but these are known to be computationally intensive and are not currently available in all standard statistical software. We describe an alternative approach using Poisson based Generalised Linear Models (GLMs).

**Methods:**

We illustrate, through application and simulation, the Poisson approach both classically and in a Bayesian framework, in two-stage and one-stage approaches. We outline the benefits of our one-stage approach through extension to modelling treatment-covariate interactions and non-proportional hazards. Ten trials of hypertension treatment, with all-cause death the outcome of interest, are used to apply and assess the approach.

**Results:**

We show that the Poisson approach obtains almost identical estimates to the Cox model, is additionally computationally efficient and directly estimates the baseline hazard. Some downward bias is observed in classical estimates of the heterogeneity in the treatment effect, with improved performance from the Bayesian approach.

**Conclusion:**

Our approach provides a highly flexible and computationally efficient framework, available in all standard statistical software, to the investigation of not only heterogeneity, but the presence of non-proportional hazards and treatment effect modifiers.

## Background

Meta-analysis methods are used to integrate quantitative findings from a set of related research studies with the aim of providing more reliable and accurate estimates of a treatment effect [1]. Traditionally a meta-analysis requires aggregate data (AD), extracted from publications or received directly from study authors. Summary statistics (e.g. log hazard ratios) are then synthesised using a fixed or random effects meta-analysis [2], where random effects can account for between study heterogeneity in the treatment effect. Meta-regression models [3] attempt to explain this excess heterogeneity with study-level covariates. However, the use of AD to conduct a meta-analysis has inherent problems, for example, hazard ratios are not always explicitly given in publications, leading to the development of alternative techniques to extract appropriate summary statistics [4]. Despite this, even when using the methods of Parmar et al., it can still be difficult to extract hazard ratios, as shown by Riley et al. [5].

An approach often considered the *gold-standard *alternative to an AD meta-analysis is a meta-analysis of individual patient data (IPD), which utilises the raw data from each study. IPD meta-analyses have been shown to be most common when analyzing time-to-event data [6]. The benefits of conducting an IPD meta-analysis with time-to-event data include: follow-up time can be longer and more up to date, analyses can be standardised across studies, model assumptions can be checked e.g. proportional hazards, and confounders can be adjusted for. However, IPD can be difficult to obtain, and a variety of methods have been developed to undertake meta-analyses from the published literature of time-to-event data. An early proposal by Dear [7] showed how to jointly synthesise survival proportions reported at multiple times, by utilising their correlation and combining them in a multivariate meta-analysis using generalised least squares. Dear investigated only fixed effects, leading the extension of Arends et al. to incorporate random effects [8]. Techniques to extract summary statistics from published studies have also been demonstated [4] for the use in standard AD meta-analyses. Fiocco et al. recently used a Poisson correlated gamma-frailty approach to combine survival curves under heterogeniety, allowing the investigation of both between-study variance and within and between-arm correlations [9]. A frailty approach has also been implemented by Feng et al. incorporating crossed random effects using penalized quasi-likelihood under a Poisson likelihood [10]. Further extensions of AD meta-analyses include assessment of the proportional hazards assumption [9,11]

IPD meta-analyses of time-to-event data can use either a two-stage or one-stage approach. The most commonly used, the two-stage, is achieved by first fitting individual survival models to each trial. The chosen estimates of effect are then combined in a standard meta-analysis framework, now equivalent to an AD meta-analysis. In a one-stage IPD meta-analysis, patient data from all studies are analysed simultaneously within a hierarchical framework. This draws parallels with the analysis of IPD from multi-centre clinical trials, accommodating clustering within treatment centres; however, in a multi-centre trial the treatment effect is not often random, whereas in a meta-analysis it often is. This is because in a multi-centre trial we can achieve greater consistency in inclusion/exclusion criteria and other aspects of trial protocol, indicating that a fixed treatment effect is likely to be more appropriate. Senn discusses these issues in more detail [12], but we emphasise that, although random-effects models are rarely used to analyse multi-centre trials, they could also adopt the methods we present here. Indeed, published trial analysis guidelines do state: "mixed models may be used to explore heterogeneity of the treatment effect. These models consider centre and treatment-by-centre effects to be random, and are especially relevant when the number of sites is large" [13]. A range of hierarchical survival models within the Cox framework have been developed [14-17], which can effectively account for heterogeneity in treatment effect and baseline risk. However, these methods can have a high computational burden and/or rely on user-written programs, not currently available in standard statistical software [16]. Furthermore, these models do not investigate the validity of the assumption of proportional hazards. These reasons serve as motivation to consider alternative approaches, such as the percentile ratio [18] as a target of inference in this setting, developed predominantly for when the proportional hazards assumption appears violated.

The aim of this paper is to explore the use of Poisson regression, and the generalised mixed model extensions, to incorporate random effects to perform one- and two-stage IPD meta-analyses of time-to-event outcomes, as an alternative to hierarchical Cox models, and to extend the models to incorporate non-proportional hazards and treatment-effect modifiers.

## Methods

### The Poisson approach to survival analysis

Poisson regression is used in the modelling of count data and contingency tables; however, the extension to modelling survival data via a piecewise exponential model [19] serves as an alternative approach to the widely used Cox model. It has been shown how the Cox model can be fitted using a Poisson GLM due to the shared form of the contribution to the partial log-likelihood, by splitting follow-up time into as many intervals as there are events [20]. However, this method can be computationally intensive. Alternatively, we can choose a smaller number of time intervals with fixed length, where patients are at risk of experiencing events within these [21], to closely approximate the Cox model. We also obtain direct estimates of the baseline hazard rate. Fine splitting of the timescale has been used to allow the use of splines and fractional polynomials to model the baseline hazard continuously [21,22].

A standard approach when choosing interval lengths is to use yearly splits [23]. The narrower the time interval, the more computationally intensive these methods will be; however, methods to compensate for this are available and described below. The shape of the underlying hazard function plays a crucial role in choosing the number of intervals necessary to successfully capture its variation. In this paper, quarter year, half year and yearly splits are compared.

Undertaking a one-stage IPD meta-analysis within a Poisson framework is beneficial due to the highly developed area of GLMs. Random effects GLMs are available within all commonly used statistical software packages (e.g. Stata, SAS and R), allowing models to be applied without the need for specialist software.

#### Model fitting in a single trial

Consider the analysis of time-to-event data from a single trial, investigating the effect of a treatment. For the *i^th ^*patient, let *x_i _*denote treatment group, coded 0/1 to denote control/treatment. A standard Cox proportional hazards model can be applied (and estimated by maximising the partial likelihood [24]):

(1) $$h_{i}\left(t\right)=h_{0}\left(t\right)\text{exp}\left(\beta_{1}x_{i}\right)$$

where *h*_0_(*t*) is the unspecified baseline hazard rate and β_1 _the log hazard ratio (i.e. the treatment effect) for the treatment group compared to the control group. By splitting follow-up time into *k *= 1,...,*K *intervals and assuming a constant hazard within each interval we can apply the Poisson model:

(2) $$\begin{array}{l} \;\;\;\;d_{ik}\sim \text{Poisson}(\mu_{ik})\\ \log(\mu_{ik})=\beta_{1}x_{i}+\lambda_{k}+\log(y_{ik}) \end{array}$$

where *d_ik _*is the event indicator, taking the value of 0 or 1 (censored or event), representing a Poisson process for each patient during each time interval. Note that *d_ik _*will not follow a Poisson distribution *per se*, but by doing so we recover the correct form of the likelihood for a piecewise exponential model. β_1 _is once again the log hazard ratio for the treatment group compared to the control group. λ_*k *_is the baseline hazard rate during the *k^th ^*time interval. Time at risk, *y_ik_*, is included as a log offset in the linear predictor. If we split follow-up time at each unique event time and apply the Poisson model, we would obtain an identical estimate of the treatment effect, β_1_, to that from a Cox model.

### Two-stage IPD meta-analyses models for survival data

The two-stage approach can be thought of as more traditional, with individual survival models applied to each trial, and appropriate summary statistics extracted to allow AD meta-analysis techniques to be applied.

We extract from the *j^th ^*trial: the log hazard ratio for the treatment group compared to the control group, β^1j, and its variance V(β^1j), using either Cox or Poisson models, which can then be combined in a standard AD meta-analysis. Such AD meta-analysis models include a fixed effect model, where we assume all trials are estimating the same true treatment effect, applied for example using the inverse variance weighted method [1]. Alternatively, a random effect model can be applied where we assume that each estimate of the treatment effect comes from a distribution of treatment effects, with mean β_1 _and variance τ^2^. Following a random effect meta-analysis, a prediction interval can be calculated for the predicted treatment effect in an individual study, to help show the potential impact of between-trial heterogeneity [25,26].

### One-stage IPD meta-analyses models for survival data

We now describe one-stage IPD meta-analyses models using the framework of proportional hazards models. The following models, if fitted using the Cox proportional hazards model, correspond to those developed by Tudur-Smith et. al. [14], which are estimated by maximising the penalized partial likelihood to find the best linear unbiased predictors, from which the REML estimators of the variance components were found [27].

#### Model A: Fixed treatment effect with proportional trial effects

For the *i^th ^*patient, *i *= 1,...,*n_j_*, in the *j^th ^*trial, *j *= 1,...,*J*, the hazard function at time *t *can be written as:

(3) $$h_{ij}\left(t\right)=h_{0}\left(t\right)\text{exp}\left(\beta_{0j}+\beta_{1}x_{1ij}\right)$$

where *h*_0_(*t*) is the baseline hazard function in the reference trial (say *j *= 1, so *β*_01 _constrained to be zero). *β*_0*j *_is the proportional effect on the baseline hazard function due to the *j^th ^*trial, now *j *= 2,..., *J. x_ij _*is coded -0.5/0.5 to denote control/treatment group and *β*_1 _is the log hazard ratio for the treatment group compared to the control group, assumed equal across all trials. Model A makes the restrictive assumption that the hazard functions in all trials are proportional to a common baseline function.

The treatment group coding of -0.5/0.5 is used in all one-stage models presented in this paper. Using this coding of the treatment group indicator, we assume equal variability in the log hazard rate across trials for both treatment groups. If we chose the 0/1 coding, this imposes the restrictive assumption that the variability in the log hazard rate of the treatment group coded 0, is zero [14,28].

#### Model B: Fixed treatment effect with baseline hazard stratified by trial

In reality, the assumption that the hazard functions in all trials are proportional is likely to be inappropriate. By allowing separate baseline hazard functions for each trial we can relax this assumption, whilst still assuming proportional hazards between treatment groups within each trial. Allowing separate baseline hazards, we have:

(4) $$h_{ij}\left(t\right)=h_{0j}\left(t\right)\text{exp}\left(\beta_{1}x_{1ij}\right)$$

where *h*_0*j*_(*t*) is the baseline hazard function in the *j^th ^*trial. As in Model A, *β*_1 _represents the log hazard ratio for the treatment group compared to the control group, assumed constant across trials. No allowance for between study variation in the treatment effect is made in Models A and B.

#### Model C: Random treatment effect with proportional trial effects

Models which allow for between-trial heterogeneity in the treatment effect are now considered. The following formulations assume an underlying mean treatment effect, coming from a population of treatment effects. The hazard function for the *i^th ^*patient in the *j^th ^*trial can be written as:

(5) $$\begin{array}{c} h_{ij}\left(t\right)=h_{0}\left(t\right)\text{exp}\left(\beta_{0j}+\beta_{1j}x_{1ij}\right)\\ \beta_{1j}=\beta_{1}+b_{1j}\\ b_{1j}\sim \text{N}\left(0,\tau^{2}\right) \end{array}$$

where *h*_0_(*t*) is the baseline hazard function in the reference trial (say *j *= 1, so *β*_01 _constrained to be zero). *β*_0*j *_is the proportional effect on the baseline hazard function due to the *j^th ^*trial, now *j *= 2,...,*J. β*_1 _is now interpreted as the mean log hazard ratio for a population of treatment effects, with *b*_1*j *_the deviation of the log hazard ratio in the *j^th ^*trial from the population mean. This assumes that the *b*_1*j*_'s come from a Normal distribution with mean zero and variance τ^2^. This formulation produces a measure of the between-trial heterogeneity in the treatment effect, τ^2^.

#### Model D: Random treatment effect with baseline hazard stratified by trial

Finally, separate baseline hazards are allowed, with a random treatment effect:

(6) $$\begin{array}{c} h_{ij}\left(t\right)=h_{0j}\left(t\right)\text{exp}\left(\beta_{1j}x_{1ij}\right)\\ \beta_{1j}=\beta_{1}+b_{1j}\\ b_{1j}\sim \text{N}\left(0,\tau^{2}\right) \end{array}$$

where *h*_0*j*_(*t*) is interpreted as in Model B, with *β*_1_, *b*_i*j *_and *τ*^2 ^defined in Model C. Model D, as in Model B, assumes proportional hazards across treatment groups only within trials.

Models A to D, within a hierarchical Cox framework, were applied by Tudur-Smith et al. [14] to IPD data from 5 trials comparing 2 anti-epileptic drugs with time-to-event outcome first seizure. A total of 1225 patients were analysed. To illustrate the computational burden of hierarchical Cox models, the application of Model C took 29 hours to achieve convergence, whilst the application of Model D took 53 minutes to achieve convergence.

### The Poisson approach to one-stage IPD meta-analysis models of survival data

We now introduce Poisson based GLM formulations of the models shown above. Techniques to increase the computational efficiency of the models are described in Section titled "Model fitting" below.

#### One-stage IPD Poisson generalised linear survival models

Models A and C: Fixed/random treatment effect with proportional trial effects. For time intervals, *k *= 1,...,*K*, we now have:

(7) $$h_{0k}\left(t\right)=\lambda_{k}$$

where λ_*k *_represents the constant hazard rate in the *k^th ^*interval for the control group, in the reference trial.

Models B and D: Fixed/random treatment effect with baseline hazard stratified by trial. Models B and D are similarly altered. For trials, *j *= 1,...,*J*, and time intervals, *k *= 1,...,*K*, we can write the baseline hazard function as:

(8) $$h_{0jk}\left(t\right)=\lambda_{jk}$$

where λ_*jk *_represents the constant hazard rate in the *j^th ^*trial during the *k^th ^*time interval.

#### Model fitting

We present Model A in the form of a Poisson GLM:

(9) $$\begin{array}{l} \;\;d_{ijk}\sim \text{Poisson}(\mu_{ijk})\\ \log(\mu_{ijk})=\beta_{0j}+\beta_{1}x_{ij}+\lambda_{k}+\log(y_{ijk}) \end{array}$$

where *d_ijk _*is the event indicator, taking the value of 0 or 1 (censored or event), representing a Poisson process for each patient in each trial during each time interval. *β*_0*j *_and *β*_1 _are as in Model A, with *λ_k _*once again the hazard rate in the control group of the reference trial. Time at risk, *y_ijk_*, is included as a log offset in the linear predictor. The extension to separate trial effects can be achieved by simply replacing the linear *β*_0*j *_and *λ_k _*terms with the interaction of them, i.e. Model B.

Fixed effect Models A and B can be implemented using any GLM software package, such as glm within Stata [29]. Models C and D, with random treatment effects, can be implemented using a multilevel mixed effects Poisson regression package, such as Stata's xtmepoisson.

It is widely known that within a mixed effects framework, maximum likelihood performs poorly when estimating variance parameters when there are a small number of studies [28]. This provides motivation for considering a Bayesian approach to the models discussed above, described and undertaken in the simulation study and results sections below.

If we have *N *independent Poisson distributed random variables, each with mean *λ*, then the sum of these *N *distributions is itself a Poisson distributed random variable with mean *Nλ*. Given this condition, it is possible to 'collapse' each split dataset across covariate patterns (for example, separately collapse the dataset for males and females) [30]. A Poisson GLM model can then be fitted to the collapsed dataset, giving identical parameter estimates to a Poisson GLM fitted to the non-collapsed dataset. This process dramatically reduces computation time when datasets are large; however, is only valid when categorical covariates are used. It is not possible to collapse across covariate patterns when including truly continuous covariates.

When handling sparse event data, the situation may arise when no events occur within a split time interval. In this case, when applying the models described in this section, we obtain nuisance estimates of the baseline hazard rate for any time interval in which no events occur. This can be remedied by the merging of time intervals.

### Simulation study

To fully assess the performance of these methods a simulation study was devised. Data is simulated consisting of a random treatment effect and proportional trial effects. We investigate the impact of the number of studies and time interval length by simulating either 5, 10 or 30 trials, and applying Poisson one-stage models with time intervals of length 0.25, 0.5 or 1 year. Each trial is simulated under the following steps:

1. Generate 2000 patients; 50% assigned to treatment, 50% to control.

2. Simulate a random treatment effect (on the log scale) with mean, α = -0.4, and inherent between-trial heterogeneity, *τ *= 0.2. Therefore *β*_1 _~ N(-0.4,0.2^2^), indicating a 33.0% (95% CI: 0.8%, 54.7%) reduction in the event rate due to treatment.

3. Generate a fixed trial effect, *β*_0 _~ N(0,0.5^2^), again on the log scale.

4. Generate survival times from a Weibull distribution using a formulation proposed by Bender et al. [31]. Scale and shape parameters were defined as *λ *= 0.042 and *γ *= 1.2, respectively. These values are based on fitting a Weibull survival model to the SHEP trial. All observations are censored after 5 years. This produces a 74.8% and 82.4% survival proportion after 5 years in the control and treatment groups, respectively.

This results in 9 scenarios, in which 1000 repetitions were simulated. For each simulated dataset, Model C was applied both classically using xtmepoisson within Stata, whilst WinBUGS, through the use of winbugsfromstata[32], was used to apply the equivalent Bayesian model. Each Bayesian model was applied with a burn-in of 1000 and sample of 5000. This was deemed adequate to achieve convergence through extensive testing of the simulations. Vague priors were assigned to all parameters under the Bayesian approach. The treatment group indicator was coded -0.5/0.5.

### Extensions to the one-stage approach

#### Treatment effect modifiers

It is becoming increasingly accepted that variation in treatment effects, as a source of heterogeneity, can only be sufficiently detected and explained when IPD are available [33]. IPD allows one to examine covariates and within-trial interactions at the patient-level. In contrast, meta-regression of only AD allows one to examine study-level covariates and interactions across-trials, and this has been shown to have low power to detect true interactions between patient covariates and treatment effect [34], and may also be subject to ecological bias and study level confounding [35].

The discrimination between within-trial and across-trial treatment-covariate interactions is a current issue in IPD meta-analysis [35,36], which requires further work within the survival analysis field. Below we present a simple one-stage model which produces a weighted average of the within- and across-trial interactions, though in our applied example the within-trial interaction dominates.

##### Fixed treatment effect with separate trial effects

Let *w_ij _*be a patient-level covariate, e.g. overweight status (coded 0/1 for no/yes, see Table 1) for the *i^th ^*patient in the *j^th ^*trial. Extending Model B to incorporate a treatment-covariate interaction gives:

**Table 1:** Summary statistics for the IPD meta-analysis investigating effectiveness of anti-hypertension drugs

Trial	Total number of patients		All-Cause Deaths		Percent Overweight (%)	
	Control	Treatment	Control	Treatment	Control	Treatment
ATMH	754	785	13	9	64.24	65.69
COOP	199	150	22	20	51.25	56.00
EWPH	82	90	25	24	62.20	63.33
HDFP	2371	2427	82	81	74.02	71.86
MRC1	3445	3546	63	67	67.52	69.57
MRC2	1337	1314	156	138	61.11	60.81
SHEP	2371	2365	229	210	67.95	68.84
STOP	131	137	7	4	58.78	63.50
SYCH	1121	1239	77	56	39.77	38.66
SYSE	2285	2380	126	115	68.39	68.31

(10) $$h_{ijk}\left(t\right)=\lambda_{jk}\text{exp}\left(\beta_{1}x_{ij}+\mu w_{ij}+\gamma x_{ij}w_{ij}\right)$$

where *λ_jk _*is the baseline hazard rate during the *k^th ^*time interval in the *j^th ^*trial, β_1 _now represents the treatment effect when *w_ij _*= 0, μ is the change in the log hazard rate of the control group for a one-unit increase in *w_ij _*and γ is the change in the treatment effect for a one-unit increase in *w_ij_*.

#### Non-proportional hazards for the treatment effect

It has been shown that the benefits of a treatment can be deemed greater during the initial period of follow-up time in certain contexts [37]. In this situation, an assumption of proportional hazards for the treatment effect will be violated. In other words, a beneficial treatment effect may diminish with time. We describe a simple approach of investigating the presence of non-proportional hazards in the treatment effect, which can be extended to any covariate within the model.

##### Fixed treatment effect with separate trial effects

Extending Model B, we first dichotomise follow-up time at time *t_s_*, and define a variable, *z_ijk_*, which takes the value 0 if *t *<*t_s _*or 1 if *t *≥ *t_s_*.

(11) $$h_{ijk}\left(t\right)=\lambda_{jk}\text{exp}\left(\beta_{1}x_{ij}+\phi x_{ij}z_{ijk}\right)$$

β^1 now represents the log hazard ratio for the treatment group compared to the control group when *t *<*t_s_*, with $\hat{\phi }$ the change in the log hazard ratio when *t *≥ *t_s_*, relative to when *t *<*t_s_*. The estimated log hazard ratio for the treatment group compared to the control group when *t *≥ *t_s _*is therefore a linear combination; β^1+ϕ^. The inclusion of non-proportional hazards can be investigated using the likelihood ratio test, comparing with Model B.

This can be extended by further splitting of follow-up time; however, the time variable, *z_ijk_*, would generally be assumed to have fewer intervals than those used to model the baseline hazard rate. Extension to include a time-varying treatment effect in Models A, C and D is easily conducted.

### The hypertension data

The example dataset used to illustrate the models in this paper comes from an IPD meta-analysis investigating the effects of anti-hypertension drugs in lowering systolic and diastolic blood pressure as determinants of cardiovascular outcomes [38]. Randomised controlled trials (RCTs) were selected on the availability of IPD and the comparison of an active treatment to a placebo or control. This resulted in the inclusion of 10 trials consisting of 28,581 patients. Meta-analysis is important to summarise the average treatment effect, and any heterogeneity in the treatment effect, across these different trials, and it enables a broader assessment of the effects of hypertension treatments than is possible in a single trial alone.

Summary statistics for the time-to-event outcome all-cause death and an overweight covariate are presented in Table 1. Overweight status is a binary covariate, coded 0/1 for no/yes, dichotomising Body-Mass Index (BMI) at 25 kg/m^2^. Detailed summary statistics can be found in the original meta-analysis [38].

## Results

### Single trial application

Comparing approaches, we apply a proportional hazards model investigating the effect of the treatment. The SHEP trial is used as an example, with outcome all-cause death. Estimated hazard ratios for the treatment effect are presented in Table 2. We observe complete agreement in estimates and 95% confidence intervals across models, showing a non-statistically significant reduction of 8.7% (95% CI: -10.1%, 24.3%) in the hazard of death for patients in the anti-hypertension treatment group compared to those in the control group.

**Table 2:** Estimates of treatment effect in the SHEP trial

Method	Hazard ratio	95% CI	
Cox	0.913	0.757	1.101
Poisson (1)	0.913	0.757	1.101
Poisson (0.5)	0.913	0.757	1.101
Poisson (0.25)	0.913	0.757	1.101

### Two-stage IPD meta-analyses models for survival data

We now apply two-stage random effects meta-analyses models to the hypertension data. In the first step we compare the Cox and Poisson models to obtain the estimates of the treatment effect in each trial, β^1j and associated variance V(β^1j). The second step is then conducted using the random effects AD meta-analysis model of DerSimonian and Laird [2].

**Figure 1:**
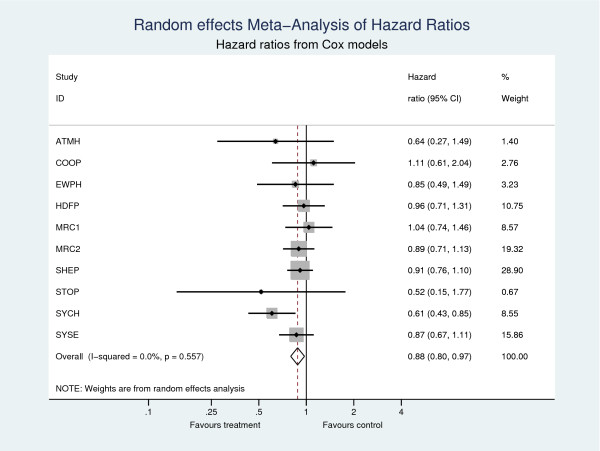
**Two-stage meta-analyses with outcome all-cause death**. Cox models are used in the first step.

**Figure 2:**
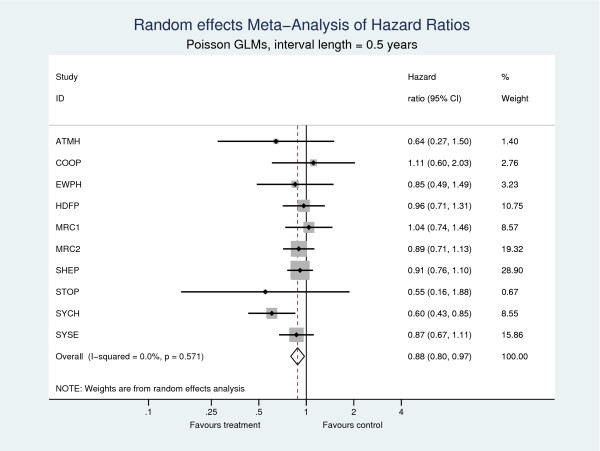
**Two-stage meta-analyses with outcome all-cause death**. Poisson GLMs are used in the first step.

Table 3 shows the estimates of the pooled hazard ratio. All 4 models produce consistent estimates of the pooled treatment effect, showing a 12% (95% CI: 2.6%, 20.4%) reduction in the hazard of death for patients in the active anti-hypertension treatment group compared to those in the control. No evidence of heterogeneity was found (τ^2=0), indicating in this case a fixed effect model would suffice and would yield identical estimates. Forest plots from the two-stage meta-analyses using Cox models and Poisson models with 0.5 year splits are shown in Figures 1 and 2, respectively, illustrating the consistent estimates of the treatment effect at both the trial and meta-analysis level.

**Table 3:** Results from two-stage random effects meta-analyses.

Model	Pooled Hazard Ratio	95% CI		τ^2
Cox	0.880	0.796	0.974	0
Poisson (0.25)	0.881	0.796	0.974	0
Poisson (0.5)	0.880	0.796	0.974	0
Poisson (1)	0.880	0.796	0.973	0

Outcome is all-cause death

### One-stage IPD meta-analyses models for survival data

We now apply each of the models described in the methods section "One-stage IPD meta-analyses models for survival data" to the hypertension data, using the Poisson method both classically and under a Bayesian approach. Further comparison of Models A(fixed treatment and fixed proportional trial effects) and B (fixed treatment and baseline stratified by trial) are made using Cox proportional hazards models, under a classical approach. Under Bayesian Models A, B, C and D all parameters are assigned a vague prior of N(0,1000^2^), excluding the heterogeneity parameter in Models C and D, where τ ~ N(0,1) with τ > 0. A burn-in of 1000 was used, with 100,000 samples and thinning at every 20th sample to remove autocorrelation.

Estimates of the treatment effect and 95% confidence/credible interval are seen in Table 4. Comparing estimates obtained under classical Cox formulations of Models A and B with equivalent Poisson models, we observe almost identical estimates of the treatment effect and 95% confidence intervals for each time interval length. For example, under all 4 classical one-stage IPD meta-analysis models with fixed treatment effect and proportional trial effects, we observe a 12.3% (95% CI: 3.0%, 20.7%) reduction in the hazard of death for patients in the active anti-hypertension treatment group compared to those in the control group. Consistent estimates of the treatment effect are obtained across all 3 choices of time interval.

**Table 4:** Estimates of the treatment effect from applying Models A to D both classically and under a Bayesian approach

Framework	Model	Treatment effect	Trial effect	Cox			Poisson (1)			Poisson (0.5)			Poisson (0.25)		
				Hazard ratio	95% CI		Hazard ratio	95% CI		Hazard ratio	95% CI		Hazard ratio	95% CI	
Classical	A	Fixed	Proportional	0.877	0.793	0.970	0.877	0.793	0.970	0.877	0.793	0.970	0.877	0.793	0.970
	B	Fixed	Stratified	0.880	0.795	0.973	0.879	0.795	0.973	0.880	0.796	0.973	0.880	0.796	0.973
	C	Random	Proportional	-	-	-	0.877	0.793	0.970	0.877	0.793	0.970	0.877	0.793	0.970
	D	Random	Stratified	-	-	-	0.879	0.795	0.973	0.880	0.796	0.973	0.880	0.796	0.973
Bayesian	A	Fixed	Proportional	-	-	-	0.877	0.796	0.971	0.878	0.792	0.969	0.876	0.792	0.970
	B	Fixed	Stratified	-	-	-	0.880	0.796	0.971	0.879	0.793	0.975	0.879	0.794	0.971
	C	Random	Proportional	-	-	-	0.874	0.756	0.994	0.871	0.747	0.994	0.873	0.748	0.998
	D	Random	Stratified	-	-	-	0.876	0.755	0.996	0.876	0.755	1.002	0.873	0.760	1.000

Each mixed effects model also produces an estimate of heterogeneity in the treatment effect, seen in Table 5. Stark contrasts in estimates of τ can be seen between classical and Bayesian approaches to both Models C and D. For example, under a classical one-stage Poisson model (with time intervals of 1 year) with random treatment effect, stratified by trial, we obtain an estimate of heterogeneity of τ = 5.92E-09 (95% CI: 0, .), compared to the equivalent Bayesian models estimate of τ = 0.081 (95% Cred. Int.: 0.004, 0.310). The classical model has estimated τ to be essentially zero, and consequently failed to construct a 95% confidence interval.

**Table 5:** Estimates of heterogeneity from applying Models C and D both classically and under a Bayesian approach

Framework	Model	Treatment effect	Trial effect	Poisson (1)			Poisson (0.5)			Poisson (0.25)		
				τ	95% CI		τ	95% CI		τ	95% CI	
Classical	C	Random	Proportional	5.83E-10	0	.	2.01E-09	0	.	5.60E-09	0	.
	D	Random	Stratified	5.92E-09	0	.	1.10E-11	0	.	4.90E-08	0	.
Bayesian	C	Random	Proportional	0.082	0.004	0.310	0.085	0.004	0.319	0.081	0.004	0.321
	D	Random	Stratified	0.081	0.004	0.310	0.080	0.004	0.299	0.077	0.003	0.306

To illustrate the computational efficiency of the method, using interval lengths of 1 year; application of Models C and D to collapsed data under a classical approach took 4.6 seconds and 60 seconds, respectively, to achieve convergence. Under a Bayesian approach the equivalent models took 64 seconds and 63 seconds, respectively, to complete the sampling.

Example code to fit Model C both classically within Stata, and under a Bayesian approach in WinBUGS [39] can be found in the Appendix.

### Simulation results

**Figure 3:**
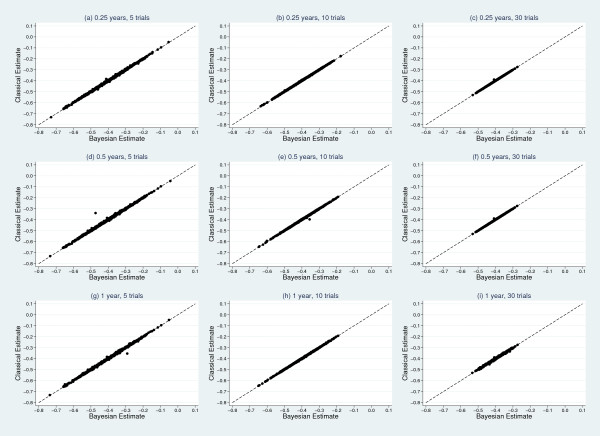
**Scatter plot matrix comparing classical and Bayesian estimates of treatment effect**. True value, α = -0.4.

**Figure 4:**
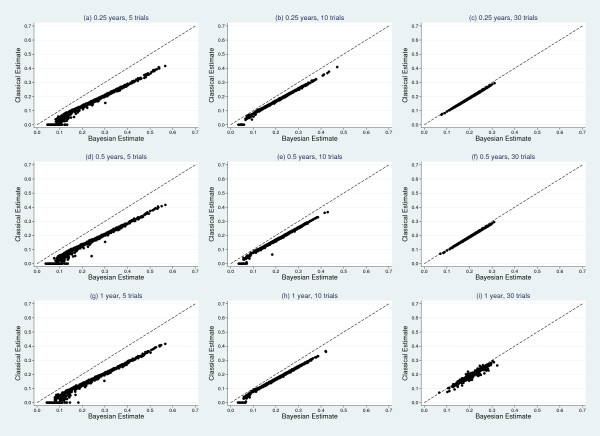
**Scatter plot matrix comparing classical and Bayesian estimates of between-study standard deviation**. True value, τ = 0.2.

Results from the simulation study, detailing mean estimates and coverages of the treatment effect and heterogeneity can be found in Tables 6 and 7, respectively. From Table 6, the treatment effect estimates appear consistent across classical and Bayesian frameworks for each model. A scatter plot matrix can be seen in Figure 3, further illustrating agreement between classical and Bayesian estimates. Coverage improves as the number of trials increase; however, within the classical models coverage is much less informative due to the moderate downward biases seen in the estimates of heterogeneity in Table 7. There is clear evidence that, irrespective of the number of trials or interval length, the classical mixed effects models consistently underestimate the true underlying heterogeneity of τ = 0.2. Estimates from the Bayesian models are generally less biased. Figure 4 shows a scatter plot matrix comparing classical and Bayesian estimates of τ, illustrating the classical approach consistently producing lower estimates of τ, compared to the Bayesian approach.

**Table 6:** Results of simulation study.

Split time	Model	5 Studies	10 Studies	30 Studies
0.25	Classical	-0.402	-0.394	-0.396
		*84.9%*	*91.4%*	*92.7%*
	Bayesian	-0.403	-0.396	-0.397
		*97.2%*	*96.2%*	*95.2%*
0.5	Classical	-0.401	-0.392	-0.396
		*84.8%*	*90.6%*	*92.7%*
	Bayesian	-0.403	-0.393	-0.397
		*97.3%*	*96.0%*	*94.7%*
1	Classical	-0.401	-0.392	-0.396
		*84.8%*	*90.7%*	*92.7%*
	Bayesian	-0.402	-0.393	-0.396
		*97.5%*	*95.6%*	*94.9%*

Bayesian estimates are means of median values. Classical estimates are mean values. True value, α = -0.4. Coverage in italics

**Table 7:** Results of simulation study.

Split time	Model	5 Studies	10 Studies	30 Studies
0.25	Classical	0.147	0.177	0.193
		-	-	*95.0%*
	Bayesian	0.230	0.213	0.205
		*95.2%*	*95.7%*	*94.3%*
0.5	Classical	0.147	0.176	0.193
		-	-	*95.0%*
	Bayesian	0.230	0.212	0.205
		*95.2%*	*94.4%*	*94.2%*
1	Classical	0.147	0.176	0.193
		-	-	*95.0%*
	Bayesian	0.231	0.212	0.207
		*95.1%*	*94.2%*	*93.9%*

Bayesian estimates are means of median values. Classical estimates are mean values. True value, τ = 0.2. Coverage in italics

We also conducted the simulations described above using a treatment group coding of 0/1. The estimates of heterogeneity from the classical model had much larger downward bias. For example, when using 0.5 year intervals, estimates of τ for 5, 10 and 30 studies were 0.112, 0.138 and 0.165, respectively when using the 0/1 treatment coding, compared with 0.147, 0.176 and 0.193 seen in Table 7 for the -0.5/0.5 coding. Estimates under a Bayesian approach remained consistent with those seen in Table 7.

We extended the simulation study to include application of Model D (random treatment effect with baseline hazard stratified by trial) to data simulated as described above. Unfortunately, due to excessive computation time, it proved infeasible to conduct the simulation study on all 9 scenarios. For example, a single run of the scenario including 10 trials with 0.25 year splits takes approximately 32 minutes. However, the 5 trial scenarios were completed and showed entirely consistent results to those described above. The computational difficulties are exclusively due to the classical approach, as each Bayesian model takes only seconds to execute the required number of MCMC samples.

### One-stage approach extensions

#### Treatment effect modifier

We apply Model (10), both classically and in a Bayesian framework, to the hypertension data to examine whether treatment effect is modified by being overweight (as defined by a BMI value ≥ 25). Note we dichotomise BMI to illustrate the methodology here, but in practice continuous variables are better analysed on their continuous scale. All parameters in the Bayesian approach use the vague prior N(0,1000^2^). Results are shown in Table 8. We observe almost identical estimates across classical models for each of the parameters of interest. When a patient is not overweight, all classical models predict a treatment effect reducing the mortality rate by approximately 14.2% (95% CI: -0.1%, 26.4.4%, 21.6%) in the hazard of death for overweight patients in the active anti-hypertension treatment group compared to those in the control. Being overweight is estimated to produce a 27.4% (95% CI: 16.5%, 37.0%) reduction in the mortality rate, with treatment group held constant. The equivalent Bayesian models produce almost identical estimates of effect compared to the classical models. Using the approach of Riley et al. [36] we also separated within-study and between-study interactions but it did not change these findings.

**Table 8:** One-stage IPD meta-analyses investigating the interaction between treatment and overweight status

Framework	Covariate	Cox			Poisson (1)			Poisson (0.5)			Poisson (0.25)		
		Hazard Ratio	95% CI		Hazard Ratio	95% CI		Hazard Ratio	95% CI		Hazard Ratio	95% CI	
Classical	Treatment when *w_ij _*= 0, exp(β^1)	0.858	0.736	1.001	0.858	0.736	1.001	0.858	0.736	1.001	0.859	0.736	1.001
	Overweight, exp(β^1)	0.726	0.630	0.835	0.725	0.630	0.835	0.726	0.630	0.835	0.726	0.630	0.835
	Treatment when *w_ij _*= 1, exp(β^1+γ^)	0.896	0.784	1.024	0.896	0.784	1.023	0.896	0.784	1.024	0.896	0.784	1.024
Bayesian	Treatment when *w_ij _*= 0, exp(β^1)	-	-	-	0.857	0.734	0.993	0.860	0.736	1.000	0.859	0.733	0.999
	Overweight, exp$\left(\hat{\mu }\right)$	-	-	-	0.725	0.634	0.836	0.726	0.632	0.838	0.725	0.631	0.840
	Treatment when *w_ij _*= 1, exp(β^1+γ^)	-	-	-	0.896	0.781	1.022	0.896	0.787	1.023	0.897	0.785	1.025

#### Non-proportional hazards

**Figure 5:**
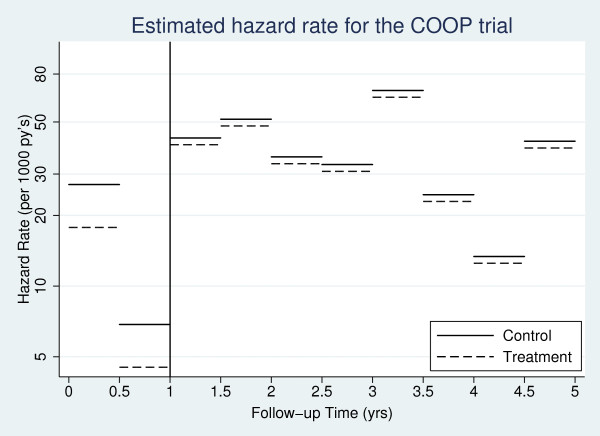
**Estimated hazard rate in the COOP trial allowing for non-proportional hazards in the treatment effect**.

We now apply Model (11) to the hypertension data, letting *t_s _*= 1. Results are presented in Table 9. From the classical models, a statistically significant (at the 5% level) 34.3% (95% CI: 16.2%, 48.5%) reduction in the hazard of death for patients in the active anti-hypertension treatment group compared to those in the control is observed in the first year of follow-up. The treatment effect after the first year is calculated by exp (β_1 _+ ϕ). This produces a non-significant reduction of 6.4% (95% CI: -4.5%, 16.2%) in the hazard of death for patients in the active anti-hypertension treatment group compared to those in the control, showing evidence of a diminishing treatment effect. Figure 5 illustrates this change by plotting the piecewise constant hazard rate in each treatment arm for the COOP trial. Extension to incorporate a random treatment effect is also possible.

**Table 9:** One-stage IPD meta-analyses investigating a non-proportional treatment effect

Framework	Covariate	Poisson (1)			Poisson (0.5)			Poisson (0.25)		
		Hazard Ratio	95% CI		Hazard Ratio	95% CI		Hazard Ratio	95% CI	
Classical	Treatment when *t *< 1, exp(β^1)	0.657	0.515	0.839	0.657	0.515	0.838	0.657	0.515	0.838
	Treatment when *t *≥ 1, exp(β^1+ϕ^)	0.935	0.837	1.045	0.936	0.838	1.045	0.936	0.838	1.045
Bayesian	Treatment when *t *< 1, exp(β^1)	0.657	0.521	0.839	0.656	0.508	0.837	0.657	0.521	0.845
	Treatment when *t *≥ 1, exp(β^1+ϕ^)	0.934	0.833	1.049	0.936	0.841	1.045	0.935	0.835	1.042

## Discussion

The importance of having IPD available has been established, allowing a full exploration of between-study heterogeneity [34] and the verification of model assumptions. By obtaining IPD, computational issues may become apparent with the sheer size of patient data being analysed when incorporating random effects. This issue is clearly highlighted when using other large datasets within the hierarchical Cox framework [14]. However, it should be noted that a variety of techniques have been developed to investigate heterogeneity and non-proportional hazards, for example, when combining aggregate level data from published studies [4,7-9,11].

In this paper, our aim was to illustrate an effective alternative to hierarchical Cox models, minimising computational issues and providing further interpretational benefits. Through minimal splitting of follow-up time, reliable estimates of effect can be obtained. Choice of interval lengths will depend on the underlying shape of the hazard function; however, the hazard ratio may be insensitive to the baseline, as illustrated by consistent estimates of the treatment effect across the 3 choices of interval length used in this paper. By combining the Poisson approach with the collapsing technique described above, we can dramatically reduce computation time. When analysing data with rare events, such models may be further advantageous through the need of less intervals. Differential follow-up times between trials can also be accounted for through this approach. Our approach provides direct estimates of the baseline hazard rate which is clinically important. These estimates allow the calculation of risk differences, or number needed to treat [40]. However, a limitation of our approach is that the collapsing technique described cannot be used with truly continuous covariates, such as age measured in days.

Investigation of random treatment effect models showed a marked underestimation of heterogeneity under the classical approach. This may in fact be explained by the tendency of maximum likelihood to underestimate variance parameters [28]. Under the Bayesian approach we observed improved performance, with comparatively lower absolute biases; however, it must be noted that, given the nature of the MCMC algorithm, the Bayesian approach will always provide a positive estimate of between study heterogeneity. A recent simulation study emphasised the need for care when choosing non-informative priors on variance parameters [41], which has specific relevance when investigating heterogeneity in the treatment effect, as in Models C and D. An alternative estimation procedure, such as h-likelihood [42], could be investigated.

It must be noted that if purely interested in a pooled treatment effect, then there is no advantage in pursuing a one-stage over a two-stage approach; however, investigation of treatment effect modifiers and modelling assumptions should be conducted simultaneously, which can only be done effectively through a one-stage approach. Although previous work has provided effective methods to investigate heterogeneity in the meta-analysis setting [14-17], we feel our approach provides a highly simplistic alternative which can incorporate the investigation of non-proportional hazards in covariate effects, and that of treatment-effect modifiers, both of which should be considered in any IPD meta-analysis.

In our analysis of the hypertension dataset, we observed a 27.4% (95% CI: 16.55, 37.0%) reduction in the mortality rate when a patient is overweight compared to a non-overweight patient, with treatment group held constant. Although this is a surprising result, it is one that has been identified previously [43]. Previous work by one of the authors of this article has also observed this relationship between BMI and mortality; however, further identified that the true factor lowering risk is height, i.e. lower risk is seen for overweight patients because they tend to be taller [44].

The approach detailed in this paper has the further benefit of allowing adjustment for confounders to be implemented simply. This becomes important when analysing IPD from observational studies, where the need to adjust for confounders is often paramount [45].

The flexibility of the Poisson approach described may be extended through the use of splines to model not only the baseline hazard, but also any time-dependent effects [21]. This would result in more plausible predictions, allowing a continuous function estimate of both.

Finally, we recognise that the IPD approach does not necessarily solve all the problems for meta-analysis [46]; in particular, IPD may not be available from all the studies requested. In this situation a sensitivity analysis may be needed to examine whether IPD meta-analysis conclusions remain robust when aggregate data from non-IPD studies are additionally included as far as possible [35].

## Conclusion

For an IPD meta-analysis of survival data, our approach provides a highly flexible and computationally efficient framework. The methods are available in all standard statistical software, allowing the investigation of not only heterogeneity, but the presence of non-proportional hazards and treatment effect modifiers.

## Competing interests

The authors declare that they have no competing interests.

## Authors' contributions

PL and RR conceived the project. MC carried out the analyses and conducted the simulation study. MC drafted the paper which was later revised by PL and RR through substantial contributions to the contents of the paper. JS, JW and FG were involved in conception, design and acquisition of the hypertension data. All authors read and approved the final manuscript.

## Appendix

### A.1. Model C: Random treatment effect with proportional trial effects

Classical model within Stata:

. *load data

. use hyperdata, clear

. *stset the data

. stset fudy, failure(death = 1) id(idnr) exit(time 5)

. *create time intervals by splitting at every year

. stsplit sp, every(1)

. egen spgrp = group(sp)

. *generate offset

. qui gen y = _t-_t0

. *collapse across covariate patterns

. collapse (min) start = _t0 (max) end = _t (count) n = _d (sum) y _d, by(spgrp treat trial)

. *fit mixed effects Poisson model with random treatment effect

. xtmepoisson _d i.treat i.trial ibn.spgrp, exposure(y) nocons irr || trial: treat, nocons

Bayesian model within WinBUGS:

model{

for (i in 1:N){

d[i] ~ dpois(mu[i]) #likelihood

log(mu[i]) < - alpha[trial[i]]*(treat[i]-0.5) + beta[trial[i]] + gamma[spgrp[i]] + log(y[i])

}

beta [1] < - 0

### Priors ###

for (s in 1:J){

alpha[s] ~ dnorm(a,tau)

}

a ~ dnorm(0,1.0E-6)

tau < - 1/var

var < - pow(sd,2)

sd ~ dnorm(0,1)I(0,)

#Trial id:

for (p in 2:J){

beta[p] ~ dnorm(0.0,1.0E-6)

}

#Intervals:

for (q in 1:ints){

gamma[q] ~ dnorm(0.0,1.0E-6)

}

### Hazard ratio due to the treatment effect:

expalpha < - exp(a)

}

## Pre-publication history

The pre-publication history for this paper can be accessed here:

http://www.biomedcentral.com/1471-2288/12/34/prepub
